# Zero-Error Coding via Classical and Quantum Channels in Sensor Networks

**DOI:** 10.3390/s19235071

**Published:** 2019-11-20

**Authors:** Wenbin Yu, Zijia Xiong, Zanqiang Dong, Siyao Wang, Jingya Li, Gaoping Liu, Alex X. Liu

**Affiliations:** 1 Jiangsu Collaborative Innovation Center of Atmospheric Environment and Equipment Technology (CICAEET), Jiangsu Engineering Center of Network Monitoring, School of Computer and Software, Nanjing University of Information Science & Technology, Nanjing 210044, China; xiongzj@nuist.edu.cn (Z.X.); 201633070012@nuist.edu.cn (S.W.); 20151243593@nuist.edu.cn (J.L.); 2 Anhui Meteorological Observatory, Hefei 230031, China; gaopingliu.ah@gmail.com; 3 Department of Computer Science and Engineering, Michigan State University, East Lansing, MI 48824-1266, USA; alexliu@cse.msu.edu; 4 The Department of Computer Science and Application, Zhengzhou Institute of Aeronautical Industry Management, Zhengzhou 450015, China; dongzanqiang2011@163.com

**Keywords:** sensor networks, communication robustness, zero-error coding, quantum channel, error correction

## Abstract

Today’s sensor networks need robustness, security and efficiency with a high level of assurance. Error correction is an effective communicational technique that plays a critical role in maintaining robustness in informational transmission. The general way to tackle this problem is by using forward error correction (FEC) between two communication parties. However, by applying zero-error coding one can assure information fidelity while signals are transmitted in sensor networks. In this study, we investigate zero-error coding via both classical and quantum channels, which consist of n obfuscated symbols such as Shannon’s zero-error communication. As a contrast to the standard classical zero-error coding, which has a computational complexity of ${O(2}^{n})$, a general approach is proposed herein to find zero-error codewords in the case of quantum channel. This method is based on a n-symbol obfuscation model and the matrix’s linear transformation, whose complexity dramatically decreases to ${O(n}^{2})$. According to a comparison with classical zero-error coding, the quantum zero-error capacity of the proposed method has obvious advantages over its classical counterpart, as the zero-error capacity equals the rank of the quantum coefficient matrix. In particular, the channel capacity can reach n when the rank of coefficient matrix is full in the n-symbol multilateral obfuscation quantum channel, which cannot be reached in the classical case. Considering previous methods such as low density parity check code (LDPC), our work can provide a means of error-free communication through some typical channels. Especially in the quantum case, zero-error coding can reach both a high coding efficiency and large channel capacity, which can improve the robustness of communication in sensor networks.

## 1. Introduction

Sensor networks are groups of specialized transducers that have a communications infrastructure that is intended to record and monitor conditions at different locations in the Internet of Things (IoT). Like any other networks, errors in messages can lead to irreversible consequences. In order to ensure the correctness of informational transmission, sensor networks should maintain a high level of robustness, security and efficiency by deploying specially designed transmission mechanisms. Some techniques have been applied to different types of sensor networks such as an efficient spider web-like transmission mechanism for emergency data in vehicular ad hoc networks [1], a spammer identification scheme based on the Gaussian mixture model that utilizes machine learning for industrial mobile networks [2] and a robustness optimization scheme to protect a class of scale-free wireless sensor networks from cyberattacks [3]. All these useful methods focus the network structure or transmission mechanism, and can improve the stabilities of sensor networks for the transmission of information. On the other hand, it is necessary to get distorted or missing information corrected actively between the senders and receivers in sensor networks. There are two basic ways to correct error information. One way is to use error control, which includes automatic repeat request (ARQ) and forward error correction (FEC). Since packets in wireless sensor networks are commonly broadcast over shared channels and forwarded over multiple hops, using FEC is preferable as it can reduce the need to retransmit data packets, thereby reducing the power consumed in the process [4]. Another way is to use error correction coding to defend noises in the transmission process.

Of course, noise always exists in channels, and finding completely noiseless channels is difficult. To address this issue, one should find a means of transmitting zero-error information. Here, it is necessary to discuss zero-error communication in sensor networks. A classical channel can be expressed as N:X→Y, where X is the set of channel inputs and Y denotes the output set. A probability transition function N(y|x) refers to the probability of the output symbol to be y when the input symbol is x. Shannon [5] initiated the study of zero-error communication, which involves transmitting messages through one and asymptotically many uses of the channel. To transmit messages through this channel with no probability of obfuscation, various messages denoted by m need to be associated with different input symbols x in such a way that the output distribution N(⋅|x) has disjoint supports. Knowledge of graph and matrix theories and combinatory and communication complexity, among others, contributes to the study of classical zero-error channels in theory. These theories and classical channel characteristics promote continued studies on zero-error communication. In a previous study [6], Lovász focused on and proposed an efficient method, namely, the Lovász ϑ function, for calculating zero-error capacity. A study was also recently conducted on the zero-error capacity of nearest-neighbor error channels with a multilevel alphabet [7].

Quantum information theory is a fascinating area that is developing rapidly and has a lot of applications in information science [8,9,10,11,12,13,14,15,16,17,18]. Qubit characteristics [19] (superposition and entangled states that cannot be cloned [20]) closely relate the quantum states to matrix and probability theories. Given its quantum characteristics, a quantum channel possesses better confidentiality and larger transmission capacity than a classical channel. Quantum zero-error communication has been increasingly becoming a subject of interest because of the compactness of zero-error communication and matrix research and the easy conversion between quantum channels and matrices. The quantum zero-error channel has attracted researchers’ attention, and quantum zero-error communication has also been studied in depth. A study [21] showed that the quantum clique problem can be defined as follows. Given a quantum channel, the number of k states that are distinguishable with no error after passing through the channel is determined. This definition reconsiders the clique problem in terms of the zero-error capacity of graphs then redefines it using quantum information theory. Super activation of the zero-error capacity of noisy quantum channels has been studied previously [22,23], and the Lovász ϑ function, which calculates quantum zero-error capacity, was introduced in previous papers [24] that featured the rewriting and deduction of the Lovász ϑ function in the quantum channel. In another work [25], R. Duan, S. Severini and A. Winter presented zero-error communication via quantum channels in the presence of noiseless feedback. Previous studies have focused on quantum zero-error capacity, with quantum zero-error coding attracting considerable attention. A quantum version of zero-error source-channel coding over a noisy channel with side information was investigated in another work [26].

This paper explores classical and quantum zero-error coding based on n-symbol obfuscation models. We introduce the classical zero-error channel model and coding method to the error correction in sensor networks, followed by a presentation of the quantum zero-error channel model. A quantum zero-error coding method based on linear transformation is proposed. Several examples are given to verify the coding efficiency and practicability of this method.

## 2. Zero-Error Coding via Classical Channel

A classical channel can be expressed as N:X→Y, where X is the set of channel inputs and Y describes the output set. A probability transition function N(y|x) denotes the probability of the output symbols to be y for input symbol x.

**Figure 1:**
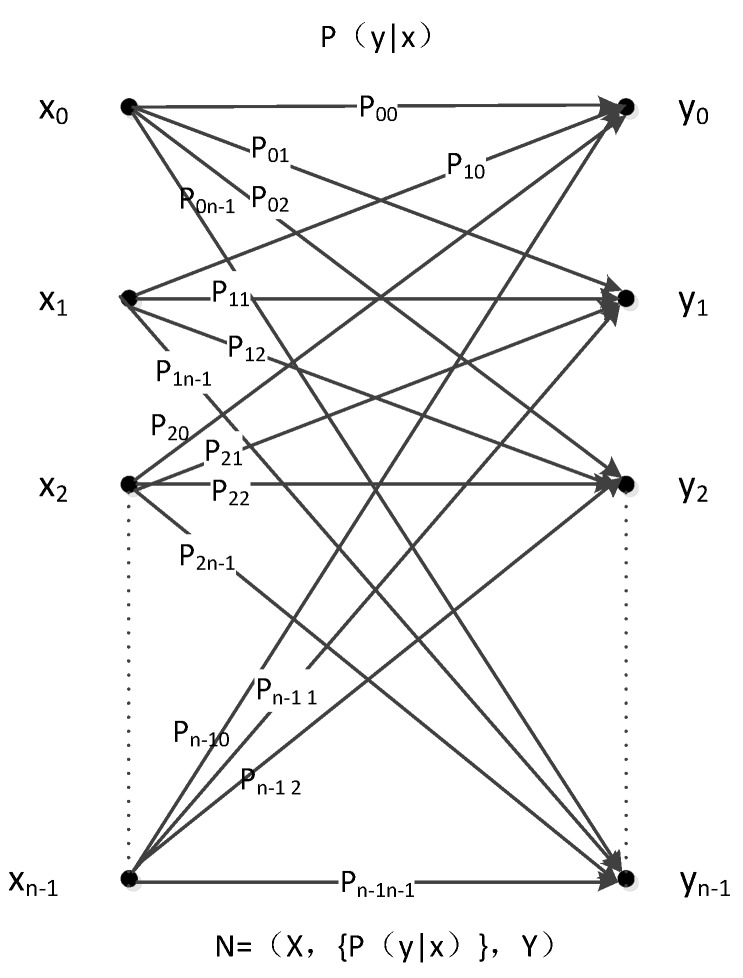
Classical channel.

Figure 1 presents the model as a channel.

In Figure 1, input set X consists of n symbols, namely, x_0_, x_1_, x_2_, ……, x_n−1_, whereas the output set also consists of n symbols, namely, y_0_, y_1_, y_2_, ……, y_n−1_. The relation between these inputs and outputs is N = (X,{P(y | x)},Y), where P_i,j_ is the probability that each input x_i_ may become output y_i_ through this channel. Next, $\sum_{j=0}^{n-1}P_{i,j}=1$ must be satisfied. In other words, if the input is x_i_, then the probability of output y_0_ is P_i,0_ while that of output y_1_ is P_i,1_, and so on, through this channel. It can confirm the value between P_00_ and P_n−1,n−1_. In addition, each value of P_i,j_ is larger than or equal to 0 and less than or equal to 1.

**Figure 2:**
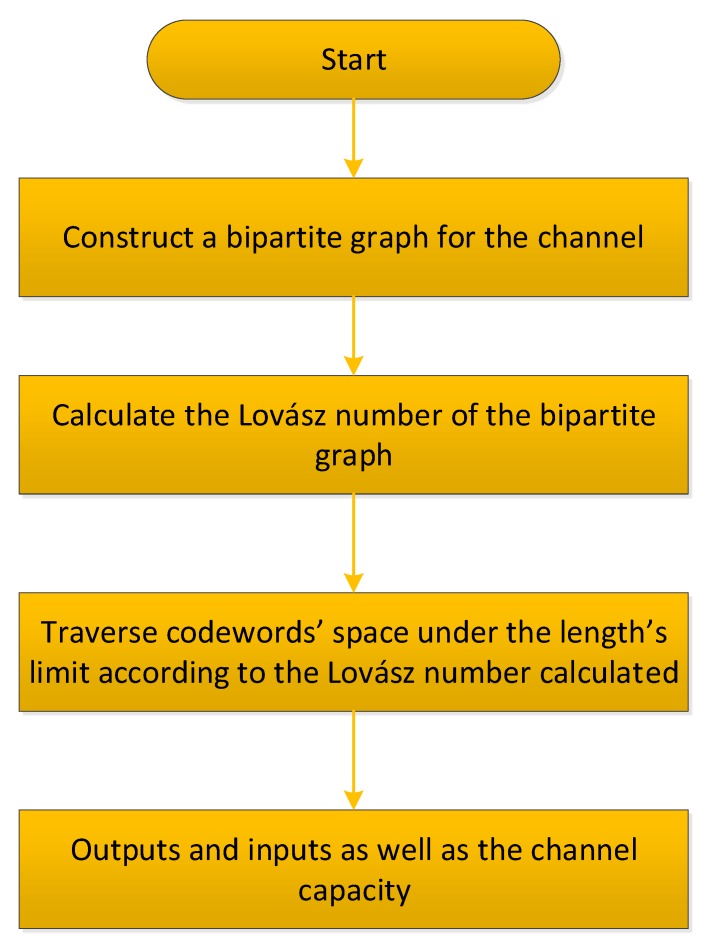
Flowchart of classical zero-error coding.

In classical communication, channel coding is achieved by combining isomorphism theory and matrix vectors. Encoding is divided into three steps. First, the channel is translated into a bipartite graph. Channels can be directly converted to bipartite graphs because probability has no effect on the computation of zero-error capacity and coding in the classical channel. Next, the Lovász ϑ function confirms the number of letters that correspond to symbols during encoding to ensure that the symbols are not confused [6]. Finally, graph isomorphism is used to determine the coding. For example, if the result of the Lovász ϑ function is calculated using $\sqrt[{3}]{n}$, then three-letter messages during encoding indicate that n symbols are non-obfuscation. The procedure for determining the n inputs is as follows. An input is assumed to be 000. Then, the disjoint output is located in 10X (X stands for any symbol of 0 or 1). If the input that matches the condition that is not confused with 000 is not found in 10X, then the input will search for 11X. These inputs are known as two non-obfuscation inputs. In this way, qualified inputs are sought constantly until the n inputs are obtained. Obviously, one almost has to traverse whole codewords’ subspaces to search for effective codewords. It will take nearly $2^{\vartheta }$ steps to complete this process. As a result, the upper bound of the total computational complexity of this method should be ${O(2}^{n})$ due to ϑ≤n. The complete procedure of the classical zero-error coding is described in Figure 2.

## 3. Zero-Error Coding via Quantum Channel

The classical zero-error channel and coding method were discussed in the previous section. This section deals with the coding problem in the quantum zero-error channel using the linear transformation of matrix.

### 3.1. Quantum n-Symbol Obfuscation Model

In quantum channels, probabilities between inputs and outputs can be represented by coefficients. Figure 2 illustrates the quantum channel.

**Figure 3:**
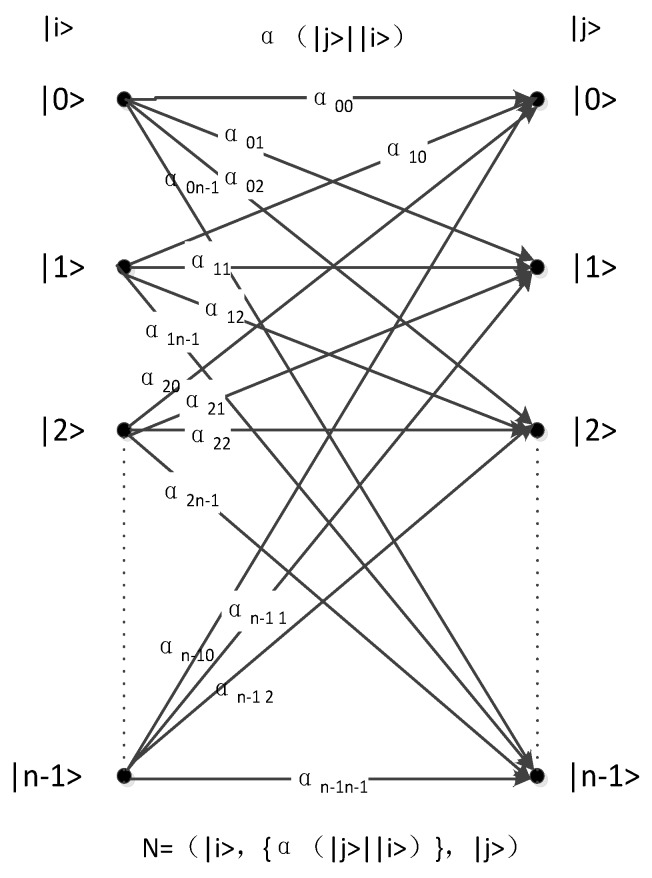
Quantum coefficient channel.

Figure 3 shows the pure states of n inputs and outputs as |0>, |1>, |2> … |n − 1>. The pure state |i> (i is an integer and i∈[0,k−1]) becomes the superposition state $|\psi >=\sum_{j=0}^{n-1}\alpha_{i,j}|j>$ via the quantum channel (ground state |j> is in the outputs; variable j is an integer and j∈[0,n−1]). $\alpha_{i,j}$ is the coefficient of the relation between ground states |i> and |j> and satisfies the condition of $\sum_{j=0}^{n-1}{\left|\alpha_{i,j}\right|}^{2}=1$. Analogous to classical channels, the probability that ground state |i> becomes ground state |j> is ${\left|\alpha_{i,j}\right|}^{2}$.

In the quantum channel, as is shown in Figure 3, the input set can be expressed as $\left\{{|x}_{i}>\right\}$, while the output set is $\left\{{|y}_{i}>\right\}$. Any input can be represented as

(1) $${|x}_{i}>=\sum_{j=0}^{n-1}b_{i,j}|j>.$$

Variable i is an integer and i∈[0,k−1]. Ground state |j> is in the outputs. Variable j is an integer and j∈[0,n−1]. $b_{\mathrm{ij}}$ is a complex number which satisfies $\sum_{j=0}^{n-1}{\left|b_{i,j}\right|}^{2}=1$.

According to quantum channel theory,

(2) $${|y}_{i}>=\frac{\sum_{j=0}^{n-1}a_{i,j}b_{i,j}|j>}{\sum_{J=0}^{n-1}{\left|a_{i,j}b_{i,j}\right|}^{2}}.$$

### 3.2. Preliminary Theorems for Zero-Error Coding in Quantum Cases

In a zero-error channel, outputs should be distinguished through coding. However, quantum states can be distinguished while they are orthogonal to each other. Considering the matrices theory, linearly independent vectors can be used to construct a set of orthogonal vectors.

Firstly, we introduce certain theorems of linear zero-error coding based on linear transformation, which is a lemma in matrices theory. Two theorems are then provided, following the lemma.

**Lemma** **1.** *The rank of a matrix is equal to the rank of its column vectors [27].*

**Proof.**  Let ${A=(\alpha }_{0}{,\alpha }_{1},\cdots {,\alpha }_{n-1})=\left[\begin{array}{cccc} \alpha_{0,0} & \alpha_{1,0} & \ldots \ldots & \alpha_{n-1,0}\\ \alpha_{0,1} & \alpha_{1,1} & \ldots \ldots & \alpha_{n-1,1}\\ \ldots \ldots & \ldots \ldots & \ldots \ldots & \ldots \ldots \\ \alpha_{0,n-1} & \alpha_{1,n-1} & \ldots \ldots & \alpha_{n-1,n-1} \end{array}\right]$ be an n × n matrix satisfying rank (A) = m. Suppose that A has an m × m submatrix $D_{m}$ with $\det\left(D_{m}\right)\neq 0$. The necessary and sufficient condition for the linear independence of a vector group $b_{1}{,b}_{2},\cdots {,b}_{m}$ is that matrix $B=\left(b_{1}{,b}_{2},\cdots {,b}_{m}\right)$, which consists of m vectors, should be of m rank. We determine that the m column in $D_{m}$ is linearly independent because $D_{m}\neq 0$. In addition, each m + 1 column vector in A is linearly dependent. Therefore, m column vectors compose the maximum linearly independent group among all column vector groups in A. Hence, the column vector groups in A are ranked m. □

**Theorem** **1.** *Let*${A=(\alpha }_{0}{,\alpha }_{1},\cdots {,\alpha }_{n-1})=\left[\begin{array}{cccc} \alpha_{0,0} & \alpha_{1,0} & \cdots & \alpha_{n-1,0}\\ \alpha_{0,1} & \alpha_{1,1} & \cdots & \alpha_{n-1,1}\\ \cdots & \cdots & \cdots & \cdots \\ \alpha_{0,n-1} & \alpha_{1,n-1} & \cdots & \alpha_{n-1,n-1} \end{array}\right]$*be an n× n matrix (rank(A) = k). Suppose that A has an n× n similarity matrix*$D=\left(c_{0}{,c}_{1},\cdots {,c}_{k-1},0,0,\cdots ,0\right)$*consisting of k linearly independent column vectors in A, such as*$c_{0}{,c}_{1},\cdots {,c}_{k-1}$*and n–k zero column vectors. Then, an n× n matrix*$B=\left[\begin{array}{cccc} b_{0,0} & b_{0,1} & \cdots & b_{0,n-1}\\ b_{1,0} & b_{1,1} & \cdots & b_{1,n-1}\\ \cdots & \cdots & b_{i,j} & \cdots \\ b_{n-1,0} & b_{n-1,1} & \cdots & b_{n-1,n-1} \end{array}\right]$*occurs, which makes*AB=D.

**Proof.**  Matrix A can be transformed into matrix D through the elementary transformation of the matrix, because D is a similarity matrix of A.

An elementary matrix E differs from the identity matrix via a single elementary row operation. Right multiplication (post-multiplication) by an elementary matrix represents elementary column operations.

For example, right multiplication by $E_{n}\left(i,j,k\right)$ is equal to the additional transformation of the column operation on matrix A, which adds column i multiplied by scalar k to column j.

Here, $E_{n}\left(i,j,k\right)=\left[\begin{array}{ccccccc} 1 & & & & & & \\ & \cdots & & & & & \\ & & 1 & \cdots & k & & \\ & & & \cdots & \cdots & & \\ & & & & 1 & & \\ & & & & & \cdots & \\ & & & & & & 1 \end{array}\right]\begin{array}{c} \leftarrow i\;\mathrm{column}\\ \leftarrow j\;\mathrm{column} \end{array}$.

In the elimination process, the matrix can be reduced step by step to matrix D. The first step is to reduce the value of $\alpha_{1,0}$ to 0. That is to say, the first column multiplying $-\frac{\alpha_{1,0}}{\alpha_{0,0}}$ is added to the second column (i.e., ${\mathrm{AB}}_{1}=A^{\prime }$) while the elements of the first row and the second column are 0s. Therefore, $B_{1}=\left[\begin{array}{ccccccc} 1 & -\frac{\alpha_{1,0}}{\alpha_{0,0}} & & & & & \\ & \cdots & & & & & \\ & & 1 & & & & \\ & & & \cdots & & & \\ & & & & 1 & & \\ & & & & & \cdots & \\ & & & & & & 1 \end{array}\right]$.

Matrix A can be transformed into matrix D through finite right multiplication by the elementary matrices $B_{1}{,\;B}_{2},\;\cdots {,\;B}_{m}$.

In other words, it can be expressed as ${D=\mathrm{AB}}_{1}B_{2}\cdots B_{m}$. Here, ${B=B}_{1}B_{2}\cdots B_{m}$. Therefore, a matrix B always exists and can produce AB = D. □

**Theorem** **2.** *Supposing the channel coefficient matrix A with rank (A) = k and the set of k inputs {* ${|x}_{i}>$ *},* ${|x}_{i}>=\frac{\sum_{j=0}^{n-1}b_{i,j}|j>}{\sqrt{\sum_{i=0}^{k-1}\sum_{j=0}^{n-1}{\left|b_{i,j}\right|}^{2}}}$ *. Here, the value of* $b_{i,j}$ *refers to the value of the corresponding position in matrix B. Matrix B is equivalent to matrix B in Theorem 1. The set of k outputs {* ${|y}_{i}>$ *} can then be distinguished.*

**Proof.**  Let $A=\left[\begin{array}{cccc} \alpha_{0,0} & \alpha_{1,0} & \cdots & \alpha_{n-1,0}\\ \alpha_{0,1} & \alpha_{1,1} & \cdots & \alpha_{n-1,1}\\ \cdots & \cdots & \cdots & \cdots \\ \alpha_{0,n-1} & \alpha_{1,n-1} & \cdots & \alpha_{n-1,n-1} \end{array}\right]$.

Considering the isomorphism between quantum states and vectors, the normalization of quantum states and Theorem 1, k linearly independent outputs and inputs can be found, which can be expressed in the following form.

Inputting ${|x}_{i}>$:

$${|x}_{i}>=\frac{\sum_{j=0}^{n-1}b_{i,j}|j>}{\sqrt{\sum_{i=0}^{k-1}\sum_{j=0}^{n-1}{\left|b_{i,j}\right|}^{2}}}(i\mathrm{is}\mathrm{an}\mathrm{integer}\mathrm{and}i\in [0,k-1]).$$

In accordance with Section 3.1, the input ${|x}_{i}>$ turns into the output ${|y}_{i}>$ after the signal passes through the channel. Here,

$${|y}_{i}>=\frac{\sum_{j=0}^{n-1}b_{i,j}\alpha_{i,j}|j>}{\sqrt{\sum_{i=0}^{k-1}\sum_{j=0}^{n-1}{\left|b_{i,j}\alpha_{i,j}\right|}^{2}}}.$$

In Theorem 1, $c_{0}{,c}_{1},\cdots ,c_{k-1}$ are linearly independent. Thus, the condition is satisfied by $\sum_{j=0}^{n-1}{(b}_{i,j}\alpha_{i,j}){(b}_{i^{\prime },j}\alpha_{i^{\prime },j})=0(i\neq i^{\prime })$, and therefore

$${<y}_{i}{|y}_{i^{\prime }}>=\frac{\sum_{j=0}^{n-1}b_{i,j}\alpha_{i,j}<j|}{\sqrt{\sum_{i=0}^{k-1}\sum_{j=0}^{n-1}{\left|b_{i,j}\alpha_{i,j}\right|}^{2}}}•\frac{\sum_{j=0}^{n-1}b_{i^{\prime },j}\alpha_{i^{\prime },j}|j>}{\sqrt{\sum_{i=0}^{k-1}\sum_{j=0}^{n-1}{\left|b_{i,j}\alpha_{i^{\prime },j}\right|}^{2}}}\;=\frac{\sum_{j=0}^{n-1}{(b}_{i,j}\alpha_{i,j}){(b}_{i^{\prime },j}\alpha_{i^{\prime },j})<j|j>}{\sqrt{\sum_{i=0}^{k-1}\sum_{j=0}^{n-1}{\left|b_{i,j}\alpha_{i,j}\right|}^{2}}\sqrt{\sum_{i=0}^{k-1}\sum_{j=0}^{n-1}{\left|b_{i,j}\alpha_{i^{\prime },j}\right|}^{2}}}=0(i\neq i^{\prime }).$$

It can be seen from the above equation that the inner product of any two different outputs is zero. This makes the discrimination of two quantum states possible. Therefore, we can say that the set of k outputs {${|y}_{i}>$} can be distinguished. □

### 3.3. Quantum Method

**Figure 4:**
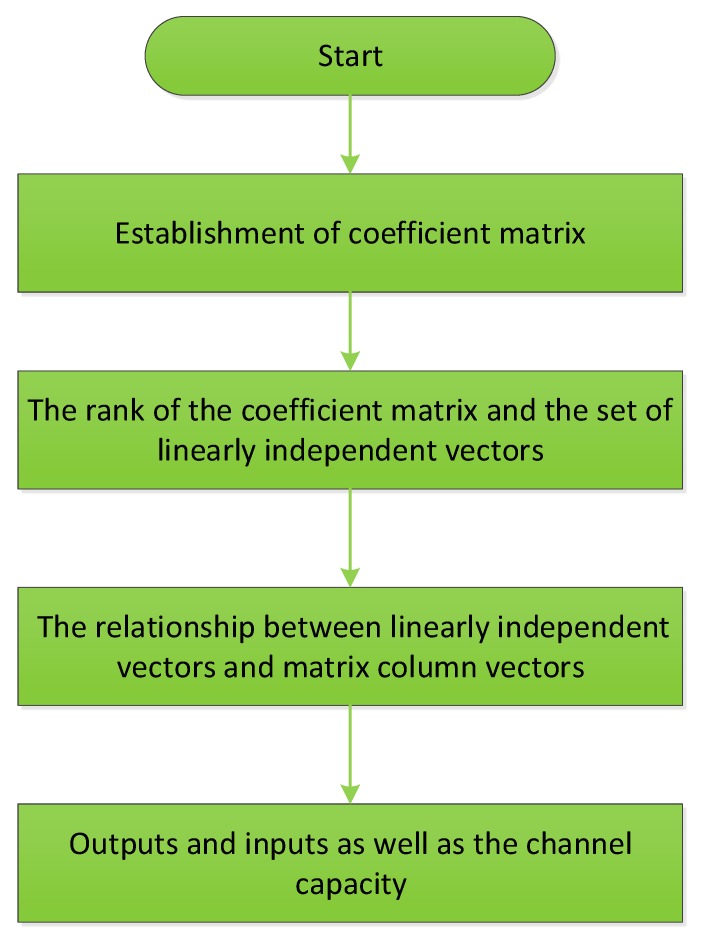
Flowchart of quantum zero-error coding based on linear transformation.

The quantum zero-error communication coding method based on linear transformation is shown in Figure 4.

Linear transformation here plays a central role in realizing the quantum zero-error coding. The following steps present the details of how it works.

A. Establishing the coefficient matrix

In the first step, the information of the quantum zero-error channel can be expressed in the form of a graph, function, or matrix depicting the relation between the ground states of n inputs and outputs. Channel matrix P and coefficient matrix A can be determined by the information of the channel, and can be represented by the following matrix:

(3) $$P=\left[\begin{array}{cccc} \alpha^{2}{}_{0,0} & \alpha^{2}{}_{1,0} & \ldots \ldots & \alpha^{2}{}_{n-1,0}\\ \alpha^{2}{}_{0,1} & \alpha^{2}{}_{1,1} & \ldots \ldots & \alpha^{2}{}_{n-1,1}\\ \ldots \ldots & \ldots \ldots & \ldots \ldots & \ldots \ldots \\ \alpha^{2}{}_{0,n-1} & \alpha^{2}{}_{1,n-1} & \ldots \ldots & \alpha^{2}{}_{n-1,n-1} \end{array}\right],$$

(4) $$A=\left[\begin{array}{cccc} \alpha_{0,0} & \alpha_{1,0} & \ldots \ldots & \alpha_{n-1,0}\\ \alpha_{0,1} & \alpha_{1,1} & \ldots \ldots & \alpha_{n-1,1}\\ \ldots \ldots & \ldots \ldots & \ldots \ldots & \ldots \ldots \\ \alpha_{0,n-1} & \alpha_{1,n-1} & \ldots \ldots & \alpha_{n-1,n-1} \end{array}\right].$$

In channel matrix P, row and column coordinates are input and output, respectively (i.e., the probability of input |i> to output |j> is $\alpha_{i,j}^{2}$). The corresponding coefficient matrix is also input to the column and row coordinates, and the expression $\alpha_{i,j}$ is the probability coefficient of input |i> to output |j>. Moreover, the column vectors in the coefficient matrix can be expressed with $\alpha_{i}={\left[\begin{array}{cccc} \alpha_{i,0} & \alpha_{i,1} & \ldots \ldots & \alpha_{i,n-1} \end{array}\right]}^{T}$.

B. The rank of the coefficient matrix and the set of linearly independent vectors

The second step is to calculate the rank k of the coefficient matrix and find the k linearly independent vectors in the coefficient matrix via matrix transformation. The set of these vectors can be expressed as $C=\left\{c_{0}{,c}_{1},\cdots {,c}_{k-1}\right\}$, with any $c_{i}$ denoted by

(5) $$c_{i}={\left[c_{i,0}{,c}_{i,1},\cdots {,c}_{i,k-1}\right]}^{T}\;(i\;\mathrm{is}\;\mathrm{an}\;\mathrm{integer}\;\mathrm{and}\;i\in [0,k-1]).$$

The rank of the coefficient matrix is k, and, in accordance with Lemma 1, the rank of the column of the matrix is also known as k. Therefore, knowing that the coefficient matrix can find at most k linearly independent column vectors is possible. These column vectors and n-k zero vectors form n × n matrix D as

(6) $$D=\left(c_{0}{,c}_{1},\cdots {,c}_{k-1},0,0,L,0\right)=\left[\begin{array}{ccccccc} c_{0,0} & c_{1,0} & \cdots & c_{k-1,\;0} & 0 & \cdots & 0\\ c_{0,1} & c_{1,1} & \cdots & c_{k-1,\;1} & 0 & \cdots & 0\\ & & \cdots & & & & \\ & & & \cdots & & & \\ & & & & \cdots & & \\ & & c_{\mathrm{ij}} & & & \cdots & \\ c_{0,n-1} & \cdots & \cdots & c_{k-1,\;n-1} & 0 & \cdots & 0 \end{array}\right].$$

C The relationship between linearly independent vectors and matrix column vectors

The third step is to determine the relation between the k linearly independent and column vectors of the coefficient matrix.

In accordance with Theorem 1, matrix B creates the following relationship between coefficient matrices A and D:

(7) AB=D.

Matrix B can be represented as

(8) $$B=\left[\begin{array}{cccc} b_{0,0} & b_{1,0} & \cdots & b_{0,n-1}\\ b_{0,1} & b_{1,1} & \cdots & b_{1,n-1}\\ \cdots & \cdots & b_{i,j} & \cdots \\ b_{0,n-1} & b_{1,n-1} & \cdots & b_{n-1,n-1} \end{array}\right].$$

By substituting Equations (4), (6) and (8) into Equation (7),

(9) $$c_{i,j}{=\alpha }_{0,j}b_{i,0}{+\alpha }_{1,j}b_{i,1}+\cdots {+\alpha }_{n-1,j}b_{i,n-1}=\sum_{q=0}^{n-1}\alpha_{q,j}b_{i,q}.$$

By substituting Equation (9) into Equation (5),

(10) $$c_{i}=\sum_{j=0}^{n-1}b_{i,j}\alpha_{j}\;(i\in [0,k-1]\;\mathrm{and}\;j\in \left[0,n-1\right]).$$

Therefore, the relation between the k linearly independent and column vectors of the coefficient matrix is Equation (10).

D Outputs and inputs as well as channel capacity

The last step is to find the outputs and inputs and the channel capacity after encoding. The encoded outputs and inputs are determined by using the isomorphism between the outputs and the linearly independent vectors. Moreover, k distinguishable outputs and their corresponding inputs can be obtained by finding the linearly independent vectors and normalization of quantum states. The outputs of the zero-error coding using the linear transformation method are distinguished by Theorem 2. Therefore, this method is appropriate.

## 4. Examples and Method Analysis

### 4.1. Pentagon Channel

**Figure 5:**
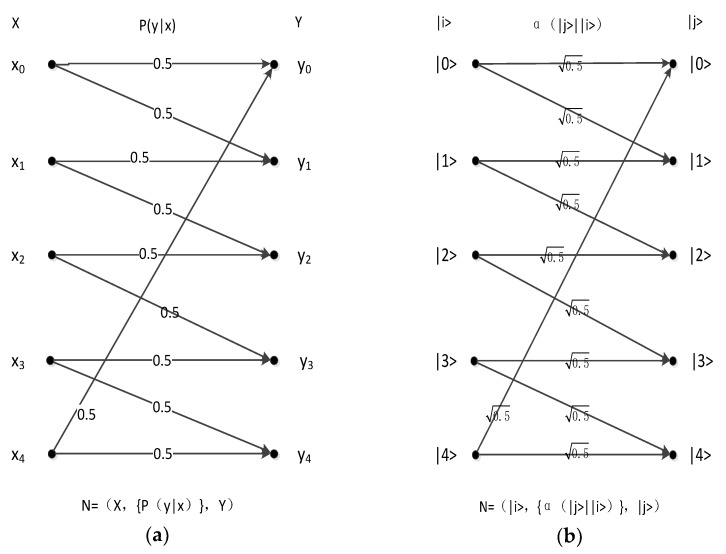
(**a**) Classical pentagonal channel; (**b**) quantum pentagonal coefficient channel.

A classic example in the study of zero-error communication is using a pentagon to represent the channel. In the study of classical zero-error communication, the coding scheme is studied in accordance with the isomorphism of graphs. In quantum zero-error communication, we can find the coding scheme through the abovementioned method. In this example, the classical and quantum coefficient channels are shown in Figure 5.

Through the classical channel, the coding {44, 32, 20, 13, 01} can realize the zero-error encoding of the classical communication. The channel capacity after encoding is $\sqrt{5}$.

If the quantum channel is replaced, then the quantum superposition principle can be used to achieve a channel capacity of 5. The coding scheme is as follows.

Inputs:

$$x_{0}=\frac{|0>-|1>+|2>-|3>+|4>}{\sqrt{5}},$$

$$x_{1}=\frac{|0>+|1>-|2>+|3>-|4>}{\sqrt{5}},$$

$$x_{2}=\frac{-|0>+|1>+|2>-|3>+|4>}{\sqrt{5}},$$

$$x_{3}=\frac{|0>-|1>+|2>+|3>-|4>}{\sqrt{5}},$$

$$x_{4}=\frac{-|0>+|1>-|2>+|3>-|4>}{\sqrt{5}}.$$

Outputs: $y_{0}=|0>$,$y_{1}=|1>$,$y_{2}=|2>$,$y_{3}=|3>$,$y_{4}=|4>$.

Authors should discuss these results and how they can be interpreted from perspective of previous studies and working hypotheses. The findings and their implications should be discussed in the broadest context possible. Future research directions may also be highlighted.

### 4.2. Triangular Channel

**Figure 6:**
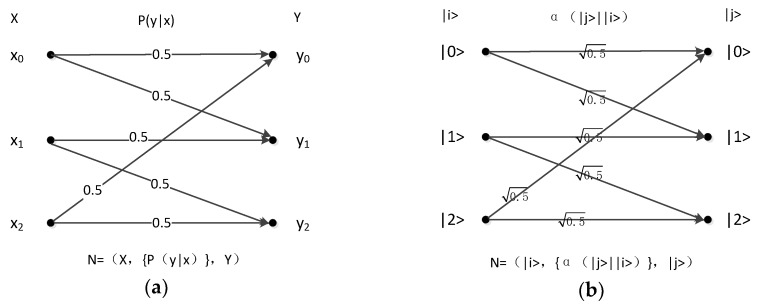
(**a**) Classical triangular channel; (**b**) quantum triangular coefficient channel.

In quantum zero-error communication, the channel is transformed into a matrix for, and the rank of the matrix is calculated. If the matrix is a full rank matrix, then the matrix elimination method can always find the linearly independent vectors with the same rank. In other words, finding the outputs of many rank numbers is always possible, as is the elimination method to determine the input. In classical zero-error communication, a superposition state is not present. Thus, the quantum zero-error capacity is larger than the classical zero-error capacity. At the same time, the zero-error capacity of the classical zero-error communication can be determined as 1 in the quantum zero-error communication. Figure 6 shows the triangular classical and quantum channels.

In classical zero-error communication, every two inputs are obfuscated. Thus, the channel is transformed into a triangle. In accordance with classical zero-error capacity theory, the capacity of this channel is 1, which is not found in the classical encoding method. In quantum zero-error communication, the rank of the coefficient matrix is 3, and the zero-error capacity is 3.

The matrix transformation method is used to solve the quantum zero-error coding. The coding scheme is as follows.

Inputs: $\frac{|0>+|2>-|1>}{\sqrt{3}}$, $\frac{|0>+|1>-|2>}{\sqrt{3}}$, and $\frac{|1>+|2>-|0>}{\sqrt{3}}$.

Outputs: |0>, |1>, and |2>.

### 4.3. Five-Symbol Multilateral Obfuscation Channel

**Figure 7:**
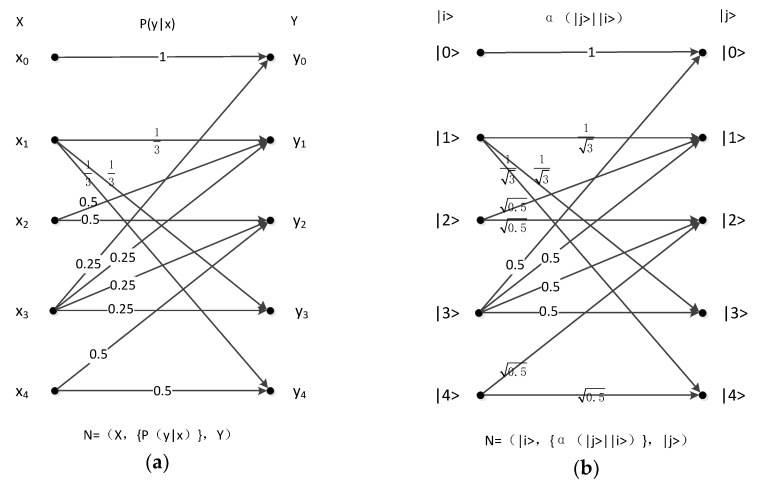
(**a**) Classical five-symbol multilateral obfuscation channel; (**b**) quantum five-symbol multilateral obfuscation channel.

Herel is a five-symbol multilateral obfuscation model, two typical examples of which are the classical channel (Figure 7a) and the quantum coefficient channel (Figure 7b) respectively.

In classical zero-error coding, we can calculate a zero-error channel capacity of 2; that is, three-letter encoding can have eight distinguishable outputs, encoding {000 001 010 011 100 101 110 111}. In quantum zero-error coding, one of the four symbols that correspond to the inputs of the four orthogonal outputs is normalized as follows.

Inputs:

$$x_{0}=|0>,x_{1}=\frac{-|0>-\sqrt{2}|2>+2|3>}{\sqrt{7}},x_{2}=\frac{-|0>-\sqrt{3}|1>+2|3>}{\sqrt{8}},\mathrm{and}x_{3}=|4>.$$

Outputs:

$$y_{0}=|0>,y_{1}=|3>,y_{2}=\frac{|2>-|4>}{\sqrt{2}},\mathrm{and}y_{3}=\frac{|2>+|4>}{\sqrt{2}}.$$

### 4.4. Performance Analysis

The zero-error capacity of the method is discussed in this section using the coding method proposed in Section 3 with several examples presented in that section.

From [5], we learn that zero-error capacity can be represented by α(G), which is the maximum number of one-letter messages that can be sent without danger of obfuscation. Therefore, the zero-error capacity of the quantum zero-error coding method based on linear transformation is the rank of the coefficient matrix, which is denoted by k. Section 3 shows that the rank in the coefficient matrix also represents the number of the linearly independent vectors of the coefficient matrix, whereas the number of linearly independent column vectors is the same as that of input symbols, which can make outputs distinguishable. When the coefficient matrix is a full rank matrix, the zero-error capacity of the n-symbol obfuscation channel is n. In this case, the method of communication can provide lossless coding. When the rank of the coefficient matrix is 1 and no two outputs are orthogonal to each other, the zero-error communication coding scheme cannot be achieved. When the rank of the coefficient matrix is k between 1 and n, there will be k orthogonal outputs and the zero-error capacity is k. It seems that n-k symbols will be discarded in orderto achieve zero-error communication. The channel capacity of this coding method is generally larger than the upper limit of the classical zero-error capacity. Moreover, in classical zero-error channels, multiple letters are often required, whereas this method requires only one letter.

In accordance with the flow of this algorithm (see Figure 3) and the formula for calculating the complexity of the algorithm, the complexity should be the sum of calculation times of each step. The number of calculation times during the first step is n, and the sum of calculation times during the second and third steps is $\frac{n(n-1)}{2}$. The number of calculation times during the final step is n. Therefore, the algorithm complexity in this paper can be expressed in the following form:

(11) $$O=O(n+\frac{n(n-1)}{2}+\frac{n(n-1)}{2}{+n)=O(n}^{2}).$$

However, the previous algorithms that solve the zero-error coding problem are all needed to traverse all subspaces in both the classical or quantum cases. The upper limit of computational complexity that traverses all subspaces is ${O(2}^{n})$. The algorithm has advantages in complexity, which is ${O(n}^{2})$ with strong feasibility.

In addition, the channel capacity and bit error rate (BER) of zero-error coding and the method of low density parity check code (LDPC) are compared as follows [28,29].

As Table 1 shows, the capacity of classical zero-error coding is much lower than its LDPC counterpart. However, the quantum capacity here looks larger than the LDPC’s since the quantum parallelism can increase channel capacity when processing qubits instead of bits.

**Figure 8:**
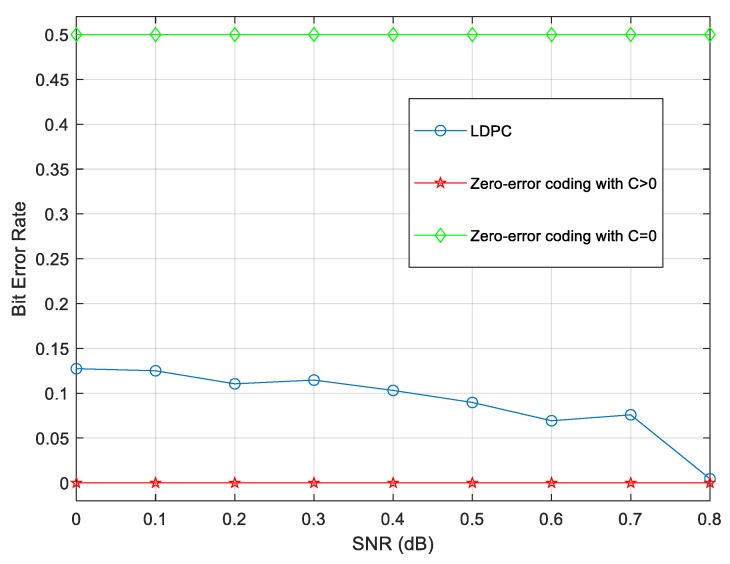
BER vs SNR in LDPC and zero-error coding cases according to channel capacity C.

Moreover, the biggest advantage of zero-error coding is that it can somehow transmit information error free on noisy channels. A comparison of LDPC and zero-error coding for both the classical and quantum cases, in terms of BER, is shown in Figure 8. When the channel capacity can maintain a non-zero positive value, even if the transmission efficiency may be lower than the LDPC case, it remains possible to communicate without any error. On the other hand, if the capacity equals 0 absolutely, there is no way to send a message to the receiver. Zero-error coding totally fails in this situation.

## 5. Conclusions

This paper presents classical and quantum zero-error coding in sensor networks, which can provide a novel way to ensure the correctness of informational transmission between senders and receivers. The classical method can obtain codewords by traversing whole subspaces with a computational complexity of ${O(2}^{n})$. The quantum method, on the other hand, is able to find linearly independent vectors through linear transformation in order to determine outputs that are orthogonal to each other, as well as column vectors, and to use the linear relationship between independent vectors to seek the corresponding input encoding. The zero-error capacity of the proposed method is the rank of the coefficient matrix in the quantum channel. We compared classical zero-error coding and the proposed quantum zero-error coding by using three examples. The comparison indicated that the proposed method has significant advantages over classical zero-error communication in terms of channel capacity. In particular, its channel capacity can reach n when the rank of coefficient matrix is full in the n-symbol multilateral obfuscation quantum channel, which cannot be reached in the classical case. Moreover, this method shows its efficiency and practicality due to the complexity of ${O(n}^{2})$. This means that, compared with traditional error-correction methods such as LDPC, our work can provide a means of error-free communication through some typical channels. Especially in the quantum case, zero-error coding was able to achieve both high coding efficiency and large channel capacity, which could improve the robustness of communication in sensor networks. What is more, tions about zero-error coding in other complex channels in sensor networks are still open, and one related to the entanglement channels is our future research interest.

## Figures and Tables

**Table 1:** The channel capacity of zero-error coding and its low density parity check code (LDPC) counterpart.

Channel Capacity C $\left(\mathrm{Calculated}\;\mathrm{in}\;Log_{2}^{N}\;\mathrm{Form}\right)$	Triangular Channel	Pentagon Channel	Five-Symbol Multilateral Obfuscation Channel
LDPC	Log(3)−1	Log(5)−1	1.405
Classical Zero-error Coding	0	$Log\left(\sqrt{5}\right)$	1
Quantum Zero-error Coding	Log(3)	Log(5)	2
